# *Aspergillus niger* Decreases Bioavailability of Arsenic(V) via Biotransformation of Manganese Oxide into Biogenic Oxalate Minerals

**DOI:** 10.3390/jof6040270

**Published:** 2020-11-09

**Authors:** Bence Farkas, Marek Kolenčík, Miroslav Hain, Edmund Dobročka, Gabriela Kratošová, Marek Bujdoš, Huan Feng, Yang Deng, Qian Yu, Ramakanth Illa, B. Ratna Sunil, Hyunjung Kim, Peter Matúš, Martin Urík

**Affiliations:** 1 Institute of Laboratory Research on Geomaterials, Faculty of Natural Sciences, Comenius University in Bratislava, 84215 Bratislava, Slovakia; farkas62@uniba.sk (B.F.); marek.bujdos@uniba.sk (M.B.); peter.matus@uniba.sk (P.M.); 2 Department of Soil Science and Geology, Faculty of Agrobiology and Food Resources, Slovak University of Agriculture in Nitra, 949 76 Nitra, Slovakia; marek.kolencik@uniag.sk; 3 Nanotechnology Centre, VŠB—Technical University of Ostrava, 70833 Ostrava, Czech Republic; gabriela.kratosova@vsb.cz; 4 Institute of Measurement Science, Slovak Academy of Sciences in Bratislava, 84104 Bratislava, Slovakia; umerhain@savba.sk; 5 Institute of Electrical Engineering, Slovak Academy of Sciences in Bratislava, 84104 Bratislava, Slovakia; edmund.dobrocka@savba.sk; 6 Department of Earth and Environmental Studies, Montclair State University, Montclair, NJ 07043, USA; fengh@montclair.edu (H.F.); dengy@montclair.edu (Y.D.); 7 School of Ecology and Environmental Science, Yunnan University, Kunming 650091, China; qianyu@ynu.edu.cn; 8 Department of Chemistry, Rajiv Gandhi University of Knowledge Technologies, AP IIIT, Nuzvid 521202, India; ramakanthilla@yahoo.com; 9 Department of Mechanical Engineering, Bapatla Engineering College, Bapatla 522101, India; bratnasunil@gmail.com; 10 Department of Mineral Resources and Energy Engineering, Jeonbuk National University, 567, Baekje-daero, Deokjin-gu, Jeonju, Jeonbuk 54896, Korea; kshjkim@jbnu.ac.kr

**Keywords:** arsenic, filamentous fungi, bioextraction, bioaccumulation, manganese minerals

## Abstract

The aim of this work was to evaluate the transformation of manganese oxide (hausmannite) by microscopic filamentous fungus *Aspergillus niger* and the effects of the transformation on mobility and bioavailability of arsenic. Our results showed that the *A. niger* strain CBS 140837 greatly affected the stability of hausmannite and induced its transformation into biogenic crystals of manganese oxalates—falottaite and lindbergite. The transformation was enabled by fungal acidolysis of hausmannite and subsequent release of manganese ions into the culture medium. While almost 45% of manganese was bioextracted, the arsenic content in manganese precipitates increased throughout the 25-day static cultivation of fungus. This significantly decreased the bioavailability of arsenic for the fungus. These results highlight the unique *A. niger* strain’s ability to act as an active geochemical factor via its ability to acidify its environment and to induce formation of biogenic minerals. This affects not only the manganese speciation, but also bioaccumulation of potentially toxic metals and metalloids associated with manganese oxides, including arsenic.

## 1. Introduction

Microbially driven processes are essential for mobilization and availability of elements in soils and sediments [1]. Filamentous fungi naturally mediate the mobilization of metals and metalloids by affecting the stability of soil particles’ and mineral surfaces via extrusion of chelating, redox active, and acidic metabolites [2], while directly disturbing the mineral structure mechanically via turgor pressure [3]. Simultaneously, however, the pre-concentration of metabolites at the cell–mineral interfaces or near the hyphae can result in formation of new thermodynamically stable biogenic minerals [4,5]. Thus, the microbially induced transformation actively contributes to immobilization of metals and metalloids in the biogenic minerals’ structure, while effectively changing the sorptive properties of these newly formed phases [6]. These processes are especially significant for environmental scavengers of elements, such as manganese oxides [7].

Manganese oxides are considered as a significant component of the natural geochemical barrier due to their ability to efficiently adsorb and immobilize metals and metalloids [4,8,9,10]. The reactive property of natural manganese oxides, including todorokite (Mn^2+^Ca, Mg)Mn^4+^_3_O_7_·H_2_O, cryptomelane K(Mn^4+^, Mn^2+^)_8_O_16_, hausmannite Mn^2+^Mn^3+^_2_O_4_ and birnessite Na_4_Mn^3+^_6_Mn^4+^_8_O_27_·9H_2_O, affects geochemical destiny of various potentially toxic metals and metalloids in the environment [11]. Hausmannite is usually found in metamorphosed or hydrothermal manganese ores, and its mineral structure is occupied by both Mn^2+^ and Mn^3+^ cations [12]. The presence of both divalent and trivalent Mn cations renders hausmannite a unique model for studying the reactivity, stability and microbially induced transformation of manganese oxides.

One of the naturally occurring hazardous elements, whose behavior is affected by redox and sorptive properties of manganese oxides [13], and which was classifies by World Health Organization (WHO) as one of the most significant environmental contaminant, is arsenic. Depending on its physical, chemical and biological factors, arsenic exists in different oxidation states −3, 0, +3, +5 [14,15,16], which significantly affect arsenic geochemical distribution and abundance, biological availability and toxicity [11,15].

As indicated, the environmental migration of arsenic is directly influenced by manganic geochemical barriers [8,9]. However, the influence of microbial activity on arsenic immobilization from manganese oxides and hydroxides is not well studied or understood. Therefore, this work focuses on evaluation of biotransformation processes occurring during mutual interaction of heterotrophic common soil fungal strain *Aspergillus niger*, arsenic and its natural scavenger and one of the rock-forming manganese oxide minerals—hausmannite. Extent and significance of microbial activity on manganese and arsenic biogeochemistry is also discussed and supported by experimental results that confirm the ability of microorganisms to form new stable biogenic mineral phases and, thus, influence the mobility of arsenic in the environment.

## 2. Materials and Methods

### 2.1. Chemicals and Reagents

All reagents used in this study were of analytical grade (Na_2_HAsO_4_·7H_2_O, MnSO_4_·4H_2_O, NaOH, HCl and HNO_3_) and were obtained from Centralchem (Bratislava, Slovak Republic) or Sigma-Aldrich (Darmstadt, Germany). For inoculation, fungal growth and other cultivation related purposes, the Sabouraud Dextrose Broth and Sabouraud Dextrose Agar culture media (HiMedia, Mumbai, India) were used.

### 2.2. Preparation of Manganese Oxide

Artificial manganese oxide hausmannite (Mn^2+^Mn^3+^_2_O_4_) was synthesized by alkaline precipitation of 1 L 0.5 mol L^−1^ MnSO_4_ using 40 g of NaOH. The mixture was then kept on a rotary shaker at 100 rpm (Unimax 2010, Heidolph, Schwabach, Germany) for 24 h at 25 °C in dark, and subsequently heated under reflux for 5 h. Synthesized precipitate was cooled, filtrated, washed with redistilled water, sterilized in a hot air oven at 80 °C for 24 h and kept in a sealed plastic bottle. It was further sterilized for 1 h in a hot air oven at 95 °C right before the experiment.

### 2.3. Fungal Strain

The fungus *Aspergillus niger* strain CBS 140837 was obtained from the fungal collection of the Department of Mycology and Physiology at the Institute of Botany, Slovak Academy of Sciences. The fungal strain was maintained on the Sabouraud agar plates at 25 °C.

### 2.4. Fungal Cultivation in Presence of Manganese Oxides with Pre-Adsorbed Arsenic

All cultivation experiments using the *A. niger* strain were performed in 100 mL sterile Erlenmeyer flasks with the mixture of 50 mL Sabouraud Dextrose Broth culture medium (HiMedia, Mumbai, India) with the initial 9.1 mg L^−1^ As(V) concentration which was prepared from a stock solution of Na_2_HAsO_4_·7H_2_O diluted in redistilled water. The 0.1 mL of spore suspensions, prepared by washing the surface of a 10-day old *A. niger* culture with sterile water and diluted to approximately 10^6^ CFU mL^−1^, were transferred into growth media under aseptic conditions to inoculate the media with filamentous fungus. Prior to inoculation, the 0.25 g of synthetized manganese oxide in an 100 mL Erlenmeyer flask were sterilized by dry heating and, after sterilization, the culture medium with As(V) content was added to the sterile flask with the manganese oxide and stirred on a rotatory shaker for 24 h at 130 rpm (Unimax 2010, Heidolph, Schwabach, Germany). There were also manganese oxide-free treatment and arsenic-free treatments which were performed as control experiments.

All experiments were carried out in triplicate under laboratory conditions during 25-day static cultivation that allowed fungus to reach the stationary growth phase. During the cultivation, the pH of culture medium, biomass weight, as well as dissolved, coprecipitated and bioaccumulated arsenic and manganese were determined on 3rd, 5th, 10th, 15th, 20th and 25th cultivation day. To evaluate arsenic and manganese distribution in cultivation system, the biomass grown on the surface of the culture medium was separated at the designated cultivation period, weighed and digested in the mixture of HCl and HNO_3_ for further analytical analysis. The spent medium was filtered, and the pH was recorded. The collected precipitates were also digested and along other components they were analyzed for total arsenic and manganese using flame atomic absorption spectrometry (F-AAS) and inductively coupled plasma mass spectrometry (ICP-MS), respectively. Furthermore, structural and morphological properties of mineral phases were characterized at the end of the cultivation.

### 2.5. Analytical Procedures

For determination of arsenic and manganese in the liquid medium, digested biomass and precipitated phases, ICP-MS (iCap Q, Thermo Scientific, Waltham, MA, USA) and F-AAS (AAS Perkin Elmer Model 1100, Waltham, MA, USA) were used, respectively [17,18,19,20]. The ICP-MS was in KED (He) mode with ^103^Rh as an internal standard. Calibration standards were prepared from As standard stock solution (1000 mg L^−1^, CertiPur, Merck, Darmstadt, Germany). The deuterium background was used for correction on F-AAS for manganese determination. Calibration standards were prepared from manganese standard stock solution (1000 mg L^−1^, CertiPur, Merck, Darmstadt, Germany).

For precise determination of crystal symmetry of manganese minerals, the X-ray diffractometer Bruker D8 DISCOVER equipped with an X-ray tube with a rotating Cu anode operating at 12 kW (40 kV/300 mA) was applied. All measurements were performed in parallel beam geometry with a parabolic Goebel mirror in the primary beam. The diffraction patterns in the angular range 20–80° of 2θ were recorded in a grazing incidence set-up with the angle of incidence α = 1.5°. A parallel plate collimator with the angular acceptance at 0.35° was inserted in the diffracted beam.

For morphology determination and particle size distribution, a Scanning Electron Microscope (SEM) QUANTA 450 FEG (FEI Company, Hillsboro, OR, USA) equipped with an Energy Dispersive Spectrometer (EDS) was used. Analysis was done at an accelerating voltage of 15 keV, and the samples were covered by a layer of carbon for better sample conduction.

Spatial distribution of newly formed thermodynamically stable biominerals incorporated in biomass were observed by 3D X-ray microscopy and computed microtomography (microCT). For the analysis, the microtomograph Nanotom 180 (GE Phoenix, Wunstorf, Germany) was used at the Institute of Measurement Science, Slovak Academy of Sciences. Nanotom 180 is equipped with point source of X-ray radiation with nano-focusation (transmission tungsten target); maximum acceleration voltage is 180 kV and energy 15 W. The applied scintillation type detector (CsI) with a matrix photodetector has image resolution of 2300 × 2300 pixels, and the size of one pixel is 50 × 50 µm. Minimal achieved voxel resolution after 3D reconstruction is down to 0.5 µm. During the measurements, an acceleration voltage of 150 kV and a current of 90 mA were applied, the detector integration time was set to 500 ms.

The determined concentrations of arsenic and manganese in biomass, culture media and non-dissolved residue, as well as biomass dry weight and culture media pH recorded during the cultivation were compared among the treatments using two-sample *t*-test assuming unequal variances in an extension Analysis ToolPak in Microsoft Excel (Redmond, WA, USA).

## 3. Results

### 3.1. Characterization of Synthesized Manganese Oxide

**Figure 1:**
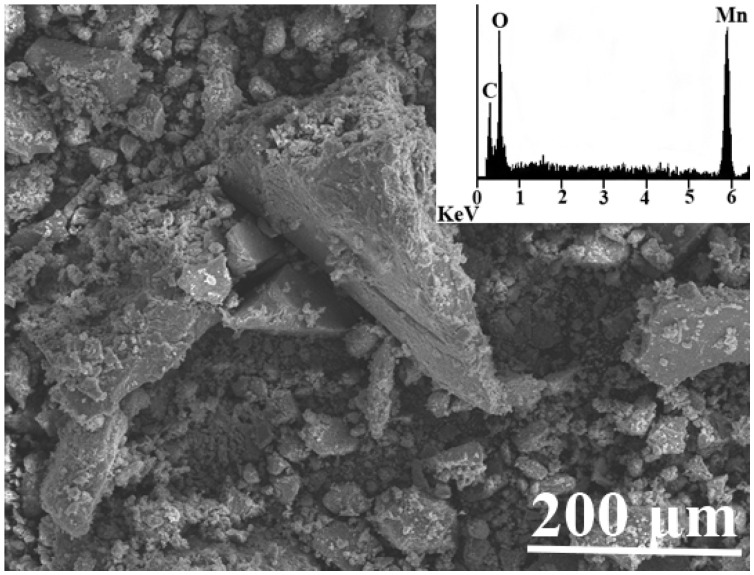
Scanning electron micrograph of manganese oxide before fungal treatment. The inset shows energy dispersive X-ray analysis (EDX) of synthesized manganese oxide.

Synthesized manganese oxide formed fine and coarse aggregates with imperfect pseudooctaedric single-grain morphology with particle size distribution between 1 µm and 200 µm. EDS chemical analysis revealed the dominant contents of manganese and oxygen (the carbon is attributed to the conductive carbon layer) and highlighted the chemical purity of crystals (Figure 1).

X-ray diffraction analysis verified tetragonal crystal symmetry with chemical formula of Mn^2+^Mn^3+^_2_O_4_, the hausmannite. Individual crystal spatial parameters such as *a*, *c*-axes are shown in Table 1.

### 3.2. Immobilization of Arsenic in the Manganese Mineral Phase(s)

**Figure 2:**
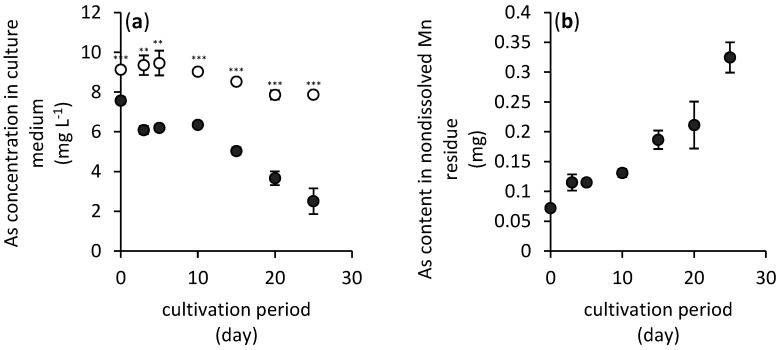
Changes in arsenic concentration in hausmannite-free (open circles) and hausmannite-treated (black solid circles) culture media supplemented with arsenic(V) (**a**) and its content in non-dissolved manganese residue (**b**) during a 25-day period of *A. niger* cultivation. Results represent the mean values of three independent experiments and error bars show the standard deviation. Asterisks indicate the significant differences between the hausmannite-free and hausmannite-treated experiments (** *p* < 0.01, *** *p* < 0.001).

The process of adsorption of dissolved arsenic(V) onto hausmannite was necessary to quantify its sorption properties. As depicted in Figure 2a, while the initial arsenic concentration in the hausmannite-free culture medium was 9.1 mg L^−1^, only 7.6 mg L^−1^ of dissolved arsenic was detected in the culture medium after 24 h pre-adsorption of arsenic(V) onto the hausmannite. Thus, the manganese oxide managed to adsorb as much as 16.4% of arsenic. This represents a 0.3 mg g^−1^ sorption capacity of hausmannite for arsenic(V).

The amount of arsenic immobilized in the non-dissolved residue of manganese, however, increased during cultivation of fungus. This is depicted in Figure 2b which shows increasing content of total arsenic bound to the manganese mineral phase(s) from initially 0.07 mg to finally 0.32 mg at the end of the fungal cultivation.

### 3.3. Manganese Bioextraction and Bioaccumulation by Fungus

**Figure 3:**
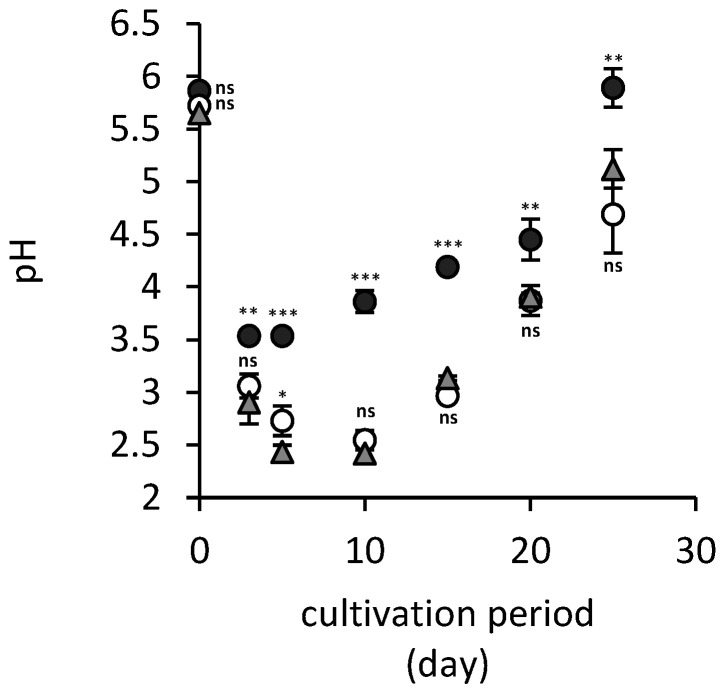
Changes in culture media pH in hausmannite-free (open circles) and hausmannite-treated (black solid circles) culture media supplemented with arsenic(V), and hausmannite- and arsenic-free controls (gray triangles) during 25-day static cultivation of an *A. niger* strain. Results represent the mean values of three independent experiments and error bars show the standard deviation. Asterisks indicate the significant differences between the hausmannite-free or hausmannite-treated experiments and controls (* *p* < 0.05, ** *p* < 0.01, *** *p* < 0.001, ^ns^ not significant).

**Figure 4:**
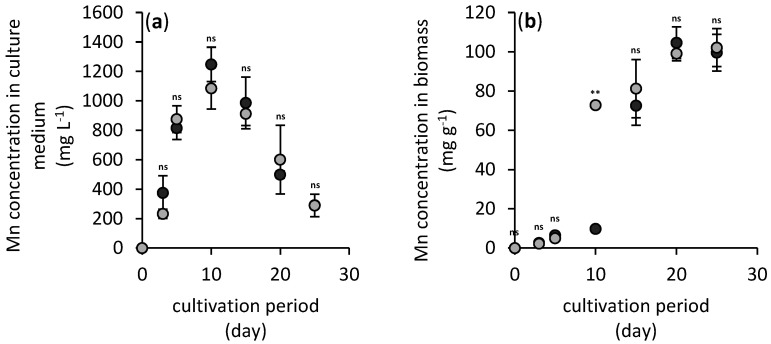
Changes in manganese concentration in arsenic-free (gray solid circles) and arsenic-treated (black solid circles) culture media (**a**) and its content in the fungal mycelium (including mineral manganese phases associated with the biomass) (**b**) during a 25-day period of *A. niger* cultivation. Results represent the mean values of three independent experiments and error bars show the standard deviation. Asterisks indicate the significant differences between the arsenic-free and arsenic-treated experiments (** *p* < 0.01, ^ns^ not significant).

During the fungal cultivation, the pH of culture media changes due to the *A. niger* strain’s acidic metabolite exudation (Figure 3). While arsenic(V) did not have any significant effect on pH development in comparison to hausmannite- and arsenic-free treatment, the presence of hausmannite and subsequent release of manganese ions (Figure 4a) resulted in statistically significantly less acidification of the culture medium by fungus. Therefore, the lowest pH values detected were 3.5 and 2.4 for manganese-treated and manganese-free culture media, respectively.

As indicated, the acidification of hausmannite-treated culture media triggered the dissolution of manganese mineral. Within the 10th day of cultivation, up to 1250 mg L^−1^ of manganese was detected in the culture medium of each treatment (Figure 4a). The presence of arsenic did not affect the dissolution rate. However, the increase in pH (Figure 3) coincides well with the significant decrease in manganese dissolved in the medium. Most of it was most likely bioaccumulated by *A. niger* (Figure 4b) since the fungal uptake resulted in manganese mycelial concentrations up to 104 mg g^−1^. However, this value also included manganese that was immobilized in newly formed biogenic minerals closely associated with fungal biomass.

During the cultivation experiment, the filamentous fungus *A. niger* was able to extract approximately 45% of manganese from its original content in hausmannite and redistribute it in the culture medium (8%) and mycelium (37%). Arsenic did not have any significant effect on the manganese redistribution in the cultivation system, and the fungus did not manage to increase the mobility of arsenic (Figure 2b).

### 3.4. Effects of Manganese Mineral Phase(s) on Arsenic Bioaccumulation

**Figure 5:**
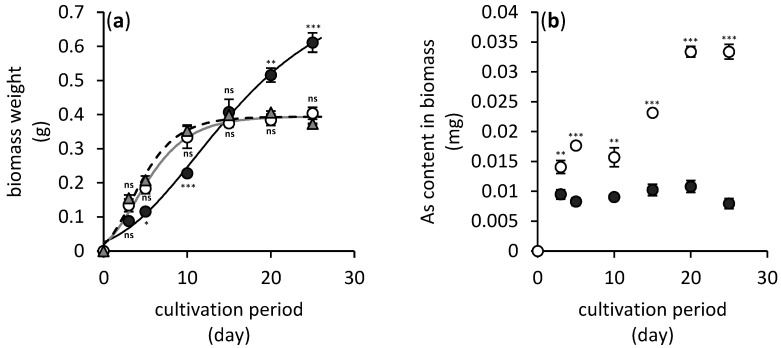
Changes in biomass dry weight (**a**) and arsenic content in biomass of fungus *A. niger* (**b**) cultivated in hausmannite-free (open circles) and hausmannite-treated (black solid circles) culture media supplemented with arsenic(V) over 25 days. Gray triangles represent hausmannite- and arsenic-free treatment. The data of *A. niger*’s biomass are fitted using modified Gompertz’s growth equation [21]. Results represent the mean values of three independent experiments and error bars show the standard deviation. Asterisks indicate the significant differences between the hausmannite-free and/or hausmannite-treated experiments and controls (* *p* < 0.05, ** *p* < 0.01, *** *p* < 0.001, ^ns^ not significant).

The fungal growth was not affected by the arsenic presence; however, manganese obviously inhibited initial growth phase of *A. niger* (Figure 5a). In the latter case, the apparent fungal biomass weight increased during the late growth phase in comparison to hausmannite-free media; however, this could be attributed to additional weight of manganese minerals intimately associated with fungal biomass.

Still, the bioavailability of arsenic in hausmannite-treated media was significantly reduced (Figure 5b). While the maximum value of accumulated arsenic was 0.035 mg in hausmannite-free media, the content of arsenic in the *A. niger* strain’s biomass did not change significantly throughout the cultivation period, and it did not exceed a value of 0.011 mg. This was the consequence of arsenic absorption in the newly formed mineral phases, or due to its adsorption onto surfaces of hausmannite or biogenic manganese minerals (Figure 2b).

### 3.5. Formation of Biogenic Manganese Oxalate

**Figure 6:**
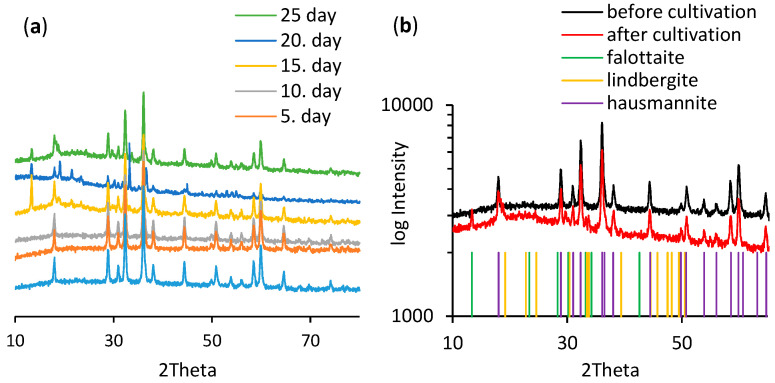
X-ray diffraction spectra of the residual solid manganese phases analyzed in pre-defined intervals (**a**). The XRD patterns indicate a transformation of the initial hausmannite into biogenic falottaite [Mn(C_2_O_4_)·3H_2_O] and lindbergite [Mn(C_2_O_4_)·2H_2_O] (**b**).

During the 25 days of *A. niger* cultivation in media supplemented with hausmannite, new biogenic precipitates were formed. The content of this new phase was after 20 days in quantities that allowed us to identify the manganese mineral falottaite [Mn(C_2_O_4_)·3H_2_O]. Later on, by the end of the cultivation experiment, it was dehydrated and partially transformed into lindbergite [Mn(C_2_O_4_)·2H_2_O]. The microbially induced transformation of hausmannite was confirmed by X-ray diffraction analysis in biomass as well as in the residual solid phase in the culture medium (Figure 6).

**Figure 7:**
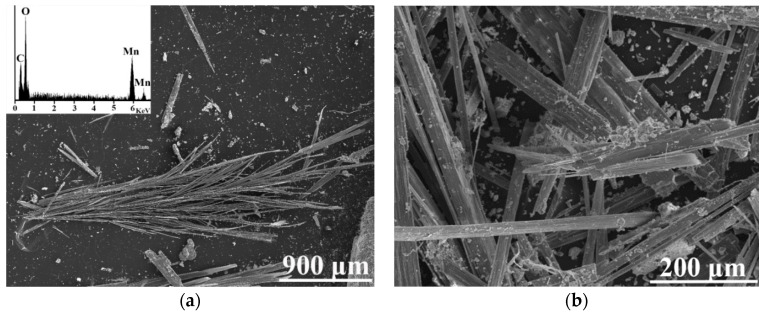
Scanning electron micrograph indicating the typical morphology of lindbergite [Mn(C_2_O_4_)·2H_2_O] (**a**) that resulted from fungal biotransformation of hausmannite [Mn^2+^Mn^3+^_2_O_4_]. Both minerals can be identified in the SEM image (**b**), where the needle-like lindbergite is associated with small grains of hausmannite.

The observed mineralogical transformation explained dynamics of manganese concentration in culture media (Figure 4a). After 10 days of cultivation, the extruded oxalate chelated dissolved manganese cations and ultimately formed biogenic manganese oxalates. The formed biogenic mineral phase lindbergite showed orthorhombic crystal symmetry with individual crystallographic parameters such as the *a*, *b, c*-axes shown in Table 2. It forms prismatic needles with long-column morphology (Figure 7a) and imperfect surfaces (Figure 7b). Its size distribution ranged from 1 µm to 1 cm.

**Figure 8:**
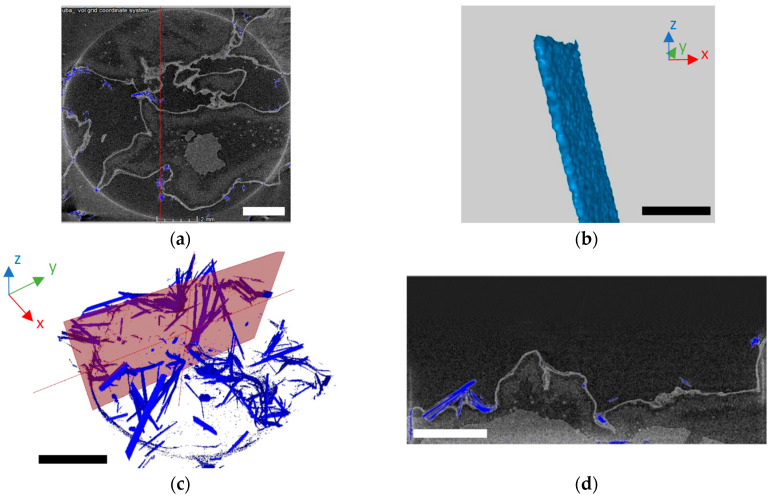
The 3D microscopy imaging of lindbergite [Mn(C_2_O_4_)·2H_2_O] crystals (indicated by blue color) that are intimately associated with fungal biomass. The general view of biomass with lindbergite (scale bar = 2 mm) (**a**); micrograph of lindbergite biogenic crystal (scale bar = 2 mm at x-axis) (**b**); lindbergite crystals associated with the mycelium visualized by 3D imaging (scale bar = 2 cm at x-axis) (**c**), and cross section of biomass with visualized lindbergite (scale bar = 1 mm) (**d**).

Formation of oxalate as a restricting mechanism for manganese availability is highlighted by the presence of biogenic manganese phases directly in fungal biomass. Lindbergite 3D visualization highlighted its spatial distribution, and surface morphology (Figure 8). It was identified in the biomass surfaces, as well as internally anchored to the biomass. While the most of the lindbergite crystals encapsulated in the biomass form smaller sized crystals with characteristic spherical and prismatic morphology, crystals situated on the biomass surfaces form larger sized crystals with needle-like morphology (Figure 8).

## 4. Discussion

The fungus *Aspergillus niger* has been shown to be capable of producing various chelating metabolites, including oxalate, citrate and various siderophores [22,23]. This enables soil minerals’ dissolution and biologically accelerated deterioration of solid surfaces in the environment [24]. Besides organic chelates, soil fungi acidify their natural habitat, which also effectively enhances the natural weathering processes [25]. Fungal acidolysis and complexolysis of solid substrates were also successfully utilized in some biohydrometallurgical methods [26,27]. These unique abilities of fungal consortia have manifested in our experiments in the potency of the fungal *A. niger* strain to decrease the pH of culture media to values as low as 2.4 (Figure 2b). However, natural soil components mitigate this effect, since we have observed that the presence of hausmannite in the culture medium alleviated the production of acidic metabolites by the fungus. Still, the acidification was strong enough to disintegrate the crystal structure of hausmannite and release almost 45% of manganese ions into the medium (Figure 4).

The proton- and ligand-mediated dissolution of manganese by filamentous fungi is well established process [28]. The excretion of oxalate has been found to be a key factor affecting the mobility of various potentially toxic metals in the environment via formation of mycogenic minerals [29,30]. In our experiments, the extrusion of oxalate by the fungus *A. niger* and bioextraction of manganese ions from hausmannite’s crystal structure has led to a precipitation and crystallization of two stable manganese oxalate minerals—falottaite and lindbergite. This process was extremely time-demanding, since the falottaite was first detected by XRD only after 15 days of cultivation (Figure 5a). Nevertheless, we suggest that both oxalates coexist, and their distribution ratio is most likely linked to changes of the culture medium pH in the late fungal growth phase.

As a significant amount of manganese oxalates were associated with the biomass (Figure 7), we hypothesize that the fragments of the fungal cell wall, or the hyphae themselves could serve as the nucleation sites via formation of saturated microdomains in the cell wall [31,32].

The outcome of hausmannite biodeterioration and transformation into mycogenic minerals have some implications not only in biogeochemistry of inorganic nutrients, but also potentially toxic metals and metalloids [6]. While we expected the decrease in arsenic content in solid manganese phases due to extensive leaching of manganese in the culture medium (Figure 4), surprisingly, biosynthesis of mycogenic mineral phases increased affinity of arsenic towards the solid surfaces and the arsenic immobilization efficiency increased during fungal cultivation (Figure 2b). It is very likely that the freshly precipitated biogenic manganese phases showed stronger affinity to adsorb and immobilize dissolved arsenic.

To conclude our experiment, we suggest that our results highlighted the significant role of microscopic filamentous fungus *A. niger* as a geoactive factor in manganese transformation and arsenic mobility. Using laboratory-based research in a model designated cultivation system, we successfully simulated the natural process of fungal interactions with a hausmannite substrate in the presence of arsenic(V). Through metabolic activity, the fungus was capable of changing the conditions in the system to an extent that resulted in manganese oxide dissolution. This led to precipitation of manganese mycogenic minerals, and subsequently affected the amount of immobilized arsenic due to changes in sorptive interactions between arsenic and the surfaces of manganese phases.

## Figures and Tables

**Table 1:** Crystallographic parameters and symmetry of synthesized manganese oxide hausmannite [Mn^2+^Mn^3+^_2_O_4_] before biotransformation.

	Manganese Oxide
crystal symmetry	tetragonal
*a* (Å)	5.763 (2)
*c* (Å)	9.459 (4)
*α* = *β* = *γ*	90°

**Table 2:** Crystallographic parameters and symmetry of biogenic lindbergite [Mn(C_2_O_4_)·2H_2_O] identified after biotransformation of hausmannite [Mn^2+^Mn^3+^_2_O_4_] by an *A. niger* strain.

	Manganese Oxalate Hydrate
crystal symmetry	orthorhombic
*a* (Å)	10.524 (2)
*b* (Å)	6.614 (2)
*c* (Å)	9.769 (3)
*α* = *β* = *γ*	90°
